# Changes in Food Choice, Taste, Desire, and Enjoyment 1 Year after Sleeve Gastrectomy: A Prospective Study

**DOI:** 10.3390/nu14102060

**Published:** 2022-05-14

**Authors:** Luigi Schiavo, Silvana Mirella Aliberti, Pietro Calabrese, Anna Maria Senatore, Lucia Severino, Gerardo Sarno, Antonio Iannelli, Vincenzo Pilone

**Affiliations:** 1 Department of Medicine, Surgery, and Dentistry, Scuola Medica Salernitana, University of Salerno, 84084 Fisciano, Italy; sialiberti@unisa.it (S.M.A.); pcalabrese@unisa.it (P.C.); asenatore@unisa.it (A.M.S.); luciaseverino1994@libero.it (L.S.); vpilone@unisa.it (V.P.); 2 Center of Excellence of Bariatric Surgery of the Italian Society of Obesity Surgery and Metabolic Disease (SICOB), Unit of General and Emergency Surgery, University Hospital San Giovanni di Dio e Ruggi d’Aragona, Mercato San Severino, 84085 Salerno, Italy; 3 General Surgery and Transplantation Unit, University Hospital San Giovanni di Dio e Ruggi d’Aragona, 84125 Salerno, Italy; gsarno79@yahoo.it; 4 Digestive Unit, Archet 2 Hospital, University Hospital of Nice, F-06202 Nice, France; iannelli.a@chu-nice.fr; 5 Inserm, U1065, Team 8, “Hepatic Complications of Obesity”, F-06204 Nice, France; 6 Faculty of Medicine, University of Nice Sophia-Antipolis, F-06107 Nice, France

**Keywords:** obesity, sleeve gastrectomy, taste, food choice, weight loss

## Abstract

Obesity is a well-recognized global health problem, and bariatric surgery (BS)-induced weight reduction has been demonstrated to improve survival and obesity-related conditions. Sleeve gastrectomy (SG) is actually one of the most performed bariatric procedures. The underlying mechanisms of weight loss and its maintenance after SG are not yet fully understood. However, changes to the taste function could be a contributing factor. Data on the extent of the phenomenon are limited. The primary objective was to assess, through validated questionnaires, the percentage of patients who report an altered perception of post-SG taste and compare the frequency of intake of the different food classes before SG and after 1 year follow-up. The secondary objective was to evaluate the total body weight change. Materials and Methods: We prospectively investigated the changes in food choice and gustatory sensitivity of 52 patients (55.8% females) 12 months after SG. The mean initial weight and body mass index (BMI) were 130.9 ± 24.7 kg and 47.4 ± 7.1 kg/m^2^, respectively. The frequency of food intake was assessed by food-frequency questionnaire, while changes in taste perception were assessed using the taste desire and enjoyment change questionnaire. The change in total body weight was also assessed. Results: A significant decrease in the intake frequency of bread and crackers (*p* < 0.001), dairy products and fats (*p* < 0.001), sweets and snacks (*p* < 0.001) and soft drinks (*p* < 0.001), and a significant increase in the frequency of vegetable and fruit consumption (*p* < 0.001) were observed at 12 months after SG in both genders. On the contrary, we found no significant changes in the frequency of meat and fish intake in females (*p* = 0.204), whereas a significant change was found in males (*p* = 0.028). Changes in perceived taste intensity of fatty foods (*p* = 0.021) and tart foods (*p* = 0.006) for females and taste of bitter foods for females and males (*p* = 0.002; *p* = 0.017) were found. Regarding the change in food desire for both genders, there was a decrease in the desire for sweet, fatty, and salty foods, whereas there was an increasing trend in the desire for tart foods, especially for females. Significant reduction in total body weight and BMI was observed in both genders at the time of follow-up. Conclusions: Based on our findings, we are able to support the evidence that changes in taste, desire, and enjoyment of taste are very common after SG, with a reduced preference for food with high sugar and fat content and an increased postoperative preference for low-sugar and -fat foods. However, further investigation is needed to clarify this issue. The molecular, hormonal, and central mechanisms underlying these changes in taste perception need to be further elucidated, as they could identify new targets able to modify obesogenic eating behavior, opening up a novel personalized therapeutic approach to obesity.

## 1. Introduction

Taste is one of the factors responsible for eating rate, as it is related to the duration of oral exposure to food, and thereby, it affects satiation [1]. The sense of taste plays an essential role in eating behavior, as it contributes to food choice, energy intake, and hence, body weight regulation, nutritional status, and health of the individual [2]. Taste variability could be the result of evolutionary adaptation mechanisms to specific environments to recognize substances potentially harmful or necessary for bodily functions [3].

It is unclear under which mechanisms taste sensitivity may influence macronutrient intake. Macronutrients interact through a variety of oral sensing mechanisms to convey signals about the quantity and quality of the ingested nutrients, contributing to the efficient metabolism and disposal of such nutrients. Understanding the range of oral sensibilities in human beings and how it is influenced by genetic and environmental variables may lead to important insights into the role of taste in food intake regulation and metabolism, and therefore, in the etiopathogenesis of obesity. The development of obesity is associated with a significant reduction in taste buds [4,5,6] and impaired taste bud sensitivity [7,8,9].

Obesity is a well-recognized global health problem, and bariatric surgery (BS)-induced weight reduction has been demonstrated to improve survival and obesity-related conditions [10]. Indeed, BS is becoming an increasingly accepted treatment for severe obesity because it results in massive and sustained weight loss with consequent improvements in health and disease outcomes [11]. Bariatric surgical procedures cause weight loss by restricting the amount of food the stomach can hold, causing malabsorption of nutrients, or by a combination of both gastric restriction and malabsorption. The most common bariatric surgery procedures are gastric bypass (GB), sleeve gastrectomy (SG), adjustable gastric band (AGB), and biliopancreatic diversion with duodenal switch (BPD/DS). In this context, SG is currently one of the most performed bariatric procedures and continues to increase worldwide because of its efficacy and low surgical risks [12,13].

After SG, patients are known to change their food preferences, suggesting a change in the underlying physiological responses to food [14]. This has been theorized as partly due to an underlying functional change, including a shift of the gut hormone profile toward an anorexic state [15,16,17]. Additionally, changes have been noted in food cravings for sweet, high-carbohydrate, and fast foods post-surgically [1,15,16,17]. Patients who had higher cravings before undergoing SG were found to have significantly decreased cravings and hedonic responses when matched with normal weight controls post-op, independent of weight loss [18]. However, to the best of our knowledge, the underlying mechanisms of weight loss and its maintenance after SG are not yet fully understood.

A few studies measured the effect of SG and other bariatric procedures on taste detection. Eight of them examined the effect of the BS on sweet taste, three on sour taste, six on salty taste, two on savory taste, and three on bitter taste [17,18,19,20,21,22,23,24]. Interestingly, studies on patients after SG have varying results. In particular, Altun et al., with the aim of assessing the changes in gustatory sensitivity of patients with obesity after SG, have shown, using standardized taste strips, for the first time in the literature, a statistically significant improvement in the taste acuity to sweet, salty, sour, and bitter at three months after SG [17]. Nance et al., with the aim of comparing the effects of SG vs. RYGB on eating behavior and sweet taste perception in patients with obesity, found that neither RYGB nor SG affected sweetness or saltiness sensitivity [23]. Furthermore, Abdeen et al., with the aim of assessing whether adolescents who undergo SG experience a change in their threshold for detecting sweet taste, show that SG did not affect the taste detection threshold for sucrose, suggesting that the shift in preference for sugary foods may be due to factors other than fundamental changes in taste sensitivity [24]. Therefore, although patients who underwent BS report changes of taste perception, results from sensory studies are discrepant and limited. Most recently, Melis et al., with the scope to determine taste function in 51 patients before, one month, and six months after undergoing BS, reported that BS can have a positive impact on gustatory functions and eating behavior, with an important role of genetic factors, which in turn might contribute to the success of the intervention [25]. Therefore, the primary objective was to assess, through validated questionnaires, the percentage of patients who report an altered perception of post-SG taste and compare the frequency of intake of the different food classes before SG and after 1 year follow-up. The secondary objective was to evaluate the total body weight change.

## 2. Materials and Methods

### 2.1. Study Design and Patients’ Selection

A single-center prospective cohort study was conducted on a cohort of patients with obesity who received SG between May and September 2020. In agreement with the interdisciplinary European guidelines on metabolic and bariatric surgery [26], inclusion criteria were a body mass index (BMI) ≥ 40 kg/m^2^ or ≥35 kg/m^2^ with obesity-related comorbidities and age between 18 and 65 years. In total, 52 patients were included in the study.

### 2.2. Endpoints

The primary objective was to assess, through validated questionnaires, the percentage of patients who report an altered perception of post-SG taste and compare the frequency of intake of the different food classes before SG and after 1 year follow-up. The secondary objective was to evaluate the total body weight change.

### 2.3. Assessment of Frequency of Food Intake and of Changes in Taste Perception

To measure the patients’ habitual food intake before and after 1 year of SG, a questionnaire was constructed by the research team based on the macro areas of the EPIC—Food Frequency Questionnaire [27] (Supplementary Material S1). To ensure reliability and validity, the questionnaire was pre-tested with a random sample of 7 patients with obesity. The results of the pre-test were not included in the study. The instrument consisted of six macro areas: (1) meat and fish (beef, pork, lamb, chicken, oily fish, fish fingers, shellfish); (2) bread and crackers (weight bread, brown bread, cheese biscuits, cream crackers); (3) dairy products and fats (cheese, salad cream, butter); (4) sweets and snacks (sweet biscuits, chocolate, cakes, ice cream, peanuts); (5) vegetables and fruits (carrots, spinach, broccoli, tomatoes, apples, pears, oranges, bananas); and (6) drinks (coffee, beer, wine, fizzy soft drink, fruit juice). Questions included an eight-point Likert scale with the end points labeled as 0 = never or less than once/month to 8 = 6^+^ times per day. A response of 0 indicated no change. The Taste Desire and Enjoyment Change Questionnaire [28] (Supplementary Material S2) was used to assess whether the degree of taste, desire, and enjoyment remained the same or had changed after undergoing SG. The questionnaire consists of 24 questions with a Likert scale where the end points are labeled 1/2 = much weaker/weaker, 3 = no change, and 4/5 = stronger/much stronger.

### 2.4. Body Weight and BMI Assessment of the Study Population

In all patients, body weight (kg) and height (cm) were determined under standard conditions (fasting state, light street clothes with shoes, and any other heavy items removed). Height was measured using a Seca 206 mechanical measuring tape (Intermed, Milano, Italy). Body weight (BW) was assessed by a Seca 869 flat digital scale (capacity 250 kg, Intermed, Milano, Italy). BW evaluation was performed at baseline and repeated by the same nutritionist during the follow-up. Body mass index (BMI) was calculated by dividing body weight (BW) by height^2^.

### 2.5. Statistical Analysis

Descriptive statistics were used to summarize patients’ characteristics, and the responses to all items were shown with absolute and relative frequencies for categorical variables and mean and standard deviation for continuous variables. Paired Student’s *t*-tests for continuous variables were performed to compare the change in food intake of female and male patients before and after the year of intervention. A response of zero in the items on change in food intake indicated no change in eating habits, so a series of one-sample *t*-tests were used to test whether the responses were significantly different from zero. A series of box plots with gender difference were used to show changes in food intake before and after one year from SG. Poisson regression analyses were performed to calculate significant predictors of taste, desire, and enjoyment on the measured variable. Incidence rate ratios (IRRs) and their 95% confidence intervals (CI) were used in the Poisson regression models to measure the independent associations between the different variables and the outcomes of interest. Multivariate analysis graphs were used to show the degree of change in taste, desire, and enjoyment between females and males (TDECQ Likert scale labels are recoded, for better understanding, as 1 = −2, 2 = −1, 3 = 0, 4 = 1, and 5 = 2). For all analyses, two-sided *p*-values of 0.05 or less were considered statistically significant. Data analyses were conducted using STATA Software (Release 16.1, StataCorp LLG, College Station, TX, USA, 2019).

## 3. Results

### 3.1. Food Intake Frequency 12 Months after Sleeve Gastrectomy

**Figure 1:**
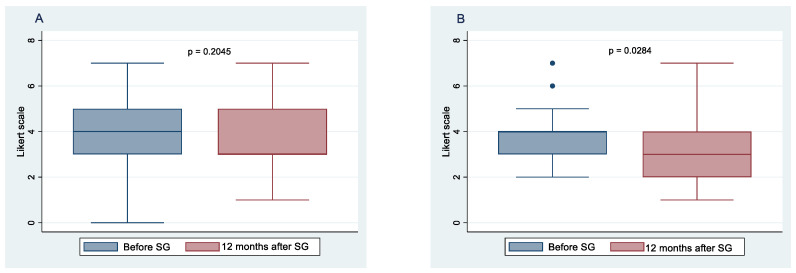

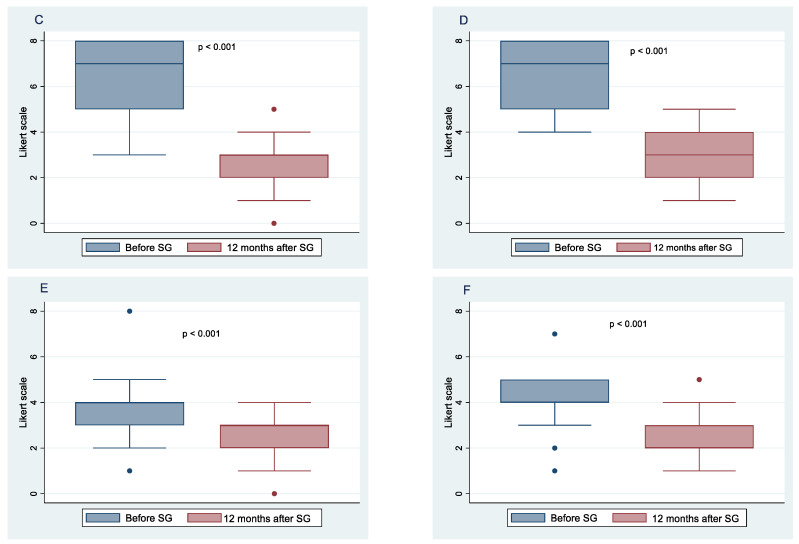
Food intake frequency before sleeve gastrectomy (SG) and after 10 months follow-up. Change in the frequency of meat and fish consumption in females (**A**), and males (**B**); change in the frequency of bread and crackers consumption in females (**C**), and males (**D**); change in the frequency of dairy products and fats consumption in females (**E**), and males (**F**).

**Figure 2:**
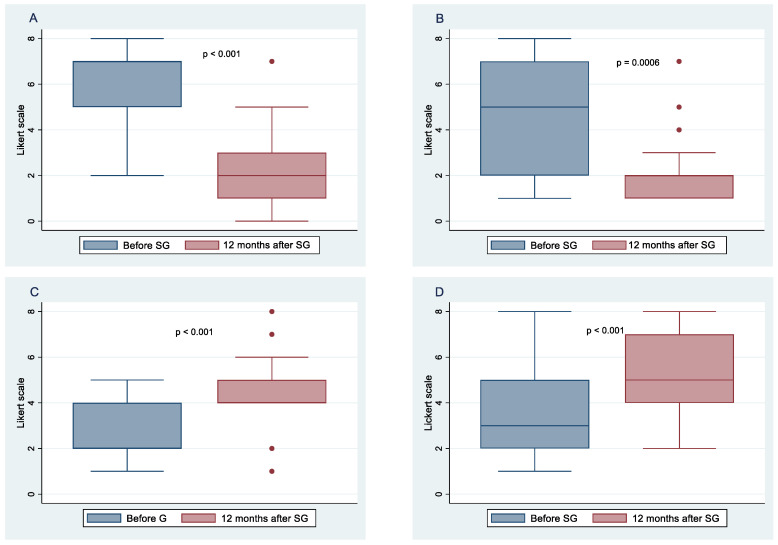

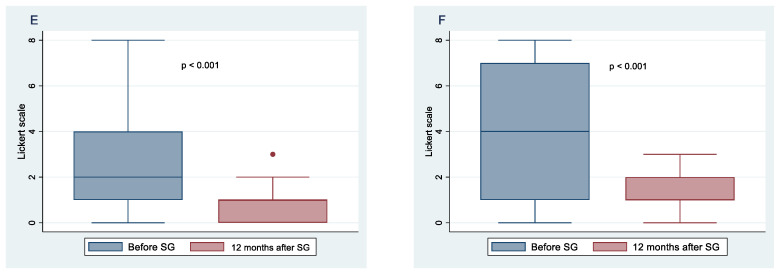
Food intake frequency before sleeve gastrectomy (SG) and after 10 months follow-up. Change in the frequency of sweet and snacks in females (**A**), and males (**B**); change in the frequency of vegetables and fruits consumption in females (**C**), and males (**D**); change in the frequency of soft drinks consumption in females (**E**), and males (**F**).

All 52 patients completed the study. Figure 1 and Figure 2 show the means and 95% confidence intervals of changes in the frequency of habitual food intake, chosen from six categories of female and male patients, respectively, before and after 12 months of SG. These results indicate statistically significant decreases for both females and males in the frequency of bread and crackers (*p* < 0.001; *p* < 0.001, respectively), dairy products and fats (*p* < 0.001; *p* < 0.001, respectively), sweets and snacks (*p* < 0.001; *p* = 0.006, respectively), and soft drinks (*p* < 0.001; *p* < 0.001, respectively). At 12 months after SG, there was a statistically significant increase in the frequency of vegetable and fruit consumption in both genders (*p* < 0.001; *p* < 0.001, respectively). There were no significant changes in the frequency of meat and fish intake between before and 1 year postoperative in females (*p* = 0.204), whereas a significant change was observed in males (*p* = 0.028).

### 3.2. Changes in Taste Perception 12 Months after Sleeve Gastrectomy

**Figure 3:**
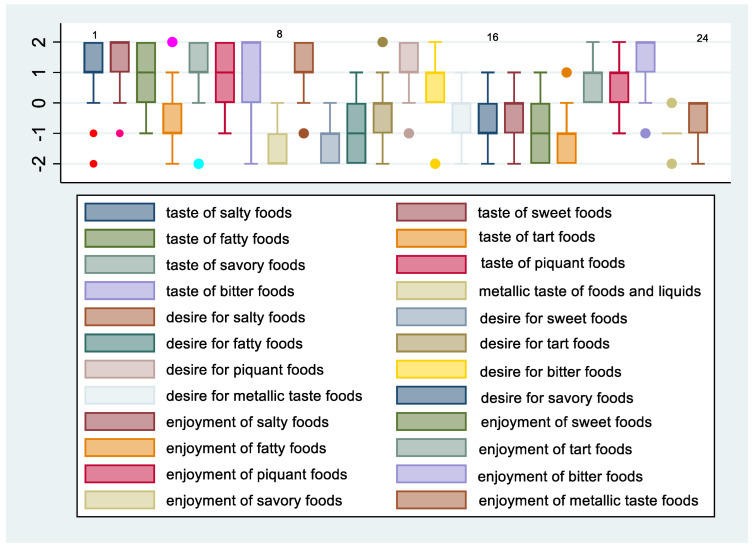
Change in taste, desire, and enjoyment 12 months after SG in females.

**Figure 4:**
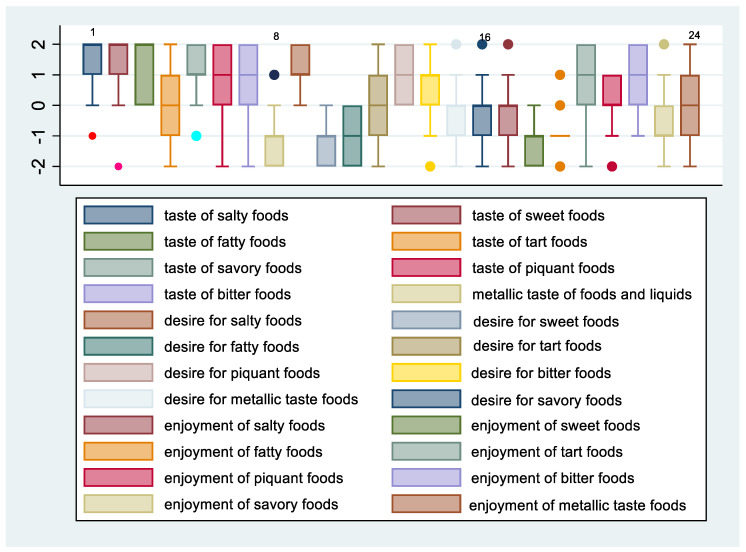
Change in taste, desire, and enjoyment 12 months after SG in males.

Figure 3 and Figure 4 show the means and 95% confidence intervals of the difference between females and males in changes in taste, desire, and enjoyment one year after SG. The first eight graphs show an increase in the intensity of the perception of some flavors, but there is no obvious gender difference between females and males in the intensity of taste perception. The Poisson regression model, constructed to study the relationship between %TWL and the change in the intensity of taste perception in females and males after SG, shows that three variables of females and one of males were statistically correlated to the outcome. These include the taste of fatty foods (IRR = 0.78, 95% CI 0.64–0.96, *p* = 0.021), tart foods (IRR = 1.45; 95% CI 1.11–1.90; *p* = 0.006), and bitter food (IRR = 1.41; 95% CI 1.13–1.77; *p* = 0.002) for females and taste of bitter food for males (IRR = 0.59; 95% CI 0.39–0.91; *p* = 0.017). Regarding the change in the desire for food flavors for both genders, there was a decrease in the desire for sweet, fatty, and salty foods, whereas there was an increasing trend in the desire for tart foods, especially for females. Poisson regression results revealed that the statistically significant predictors of %TWL were, for females, the desire for tart foods with a protective incidence ratio (IRR = 0.70; 95% CI 0.51–0.95; *p* = 0.024), while for males, the desire for bitter tastes had a protective incidence ratio (IRR = 0.66; 95% CI 0.45–0.97; *p* = 0.037). The decrease in perception of enjoyment for sweet was greater in males than in females, whereas decrease in enjoyment for fatty was greater for females than for males. There was an increase in perception of enjoyment for bitter foods for both genders but more so in females. Results from the Poisson regression model showed that by %TWL, several variables were statistically significant for females, including decreased enjoyment of sweet foods (IRR = 0.79; 95% CI 0.64–0.98; *p* = 0.035), pleasure of salty foods (IRR = 0.70; 95% CI 0.58–0.86; *p* = 0.001), pleasure of bitter foods (IRR = 0.41; 95% CI 0.21–0.78; *p* = 0.007) protective factors, whereas for males, decreased pleasure of spicy foods (IRR = 1.30; 95% CI 0.03–0.53; *p* = 0.05) was a risk factor.

### 3.3. Total Body Weight and BMI Changes 12 Months after SG

As expected, we observed significant improvement in BW and BMI at 1 year after SG (*p* < 0.001 and *p* < 0.001, respectively).

## 4. Discussion

Obesity, a chronic and progressive disease associated with significant morbidity and mortality, is affecting an increasing number of populations every year [29]. Concerning the treatment of obesity, BS has been shown to be the most effective and durable therapeutic means of long-term treatment of morbid obesity. In the field of bariatric procedures, SG, a procedure previously used as part of the duodenal switch bypass but now commonly used as a standalone procedure, is one of the most commonly performed BS procedures, and it continues to increase worldwide because of its efficacy and low surgical risks [12].

Several studies have demonstrated that SG induces a steady weight loss [30,31,32,33]. However, the exact mechanisms by which SG induces weight loss are not completely clear. While there is a general agreement that the reduction in volume of food plays a central role in post-SG weight loss [34], some studies suggest that changes in what patients eat may also contribute [1,14,15,16,17].

Obesity was shown to lower gustatory sensitivity in both children and adults, and there are studies on the preference for sweet taste in patients with obesity [4,5,6,7,8,9]. In this regard, it has been suggested that patients after SG change their preference from high to lower caloric density food [1,15,16,17]. Based on our findings, we are able to support the evidence that changes in taste, desire, and enjoyment of taste are very common after SG, with a reduced preference for food with high sugar and fat content and an increased postoperative preference for low-sugar and -fat foods.

In particular, 12 months after SG, we found a statistically significant decrease, in both genders, in the frequency of consumption of bread, crackers, dairy products, fats, sweets, snacks, and soft drinks. These data are in agreement with other studies in which the reduction in preference for sweet- and fat-containing tastes in patients with obesity after BS were found [16,17]; however, the molecular and hormonal mechanisms underlying these changes in taste perception need to be elucidated. Bariatric surgeries were shown to affect taste preferences of patients with obesity, probably due to the increase in the concentrations of anorexigenic gut hormones, which might have an effect on gustatory sensitivity [35]. In this regard, different studies have shown that the anorexigenic hormone leptin may act as a modulator of sweet taste responses in mammals, having a role in maintaining energy homeostasis [20,36,37,38]. The decrease in serum leptin was significantly associated with the decrease in the sweet taste threshold during weight loss in healthy and obese females [39].

Concerning other tastes, such as sour, salty, and bitter, herein, we found a decrease in the desire for salty foods, whereas there was an increasing trend in the desire for sour foods, especially for females. Furthermore, we found an increase in perception of enjoyment for bitter foods for both genders but more so in females. To the best of our knowledge, only a few studies are available on the relation between BS and salt perception, with inconsistent results [20,40]. The lack of changes in saltiness that we found after bariatric surgery is consistent with results showing bariatric surgery does not alter the salt detection threshold [20,22] or the hedonic responses evoked by cream soups differing in salt concentrations [22]. Furthermore, as for the sweet taste, studies observed a significant improvement in salty taste detection 1, 3, and 6 months after SG [18,22]. Moreover, concerning bitter taste, the literature includes only two studies that report a significant improvement in bitter taste detection at 1 and 3 months after SG [17,21]. Most recently, Melis et al. showed that BS is associated with an overall increase in taste sensitivity, although changes in the taste score after surgery were mostly explained by increasing identification of sweet, sour, and umami stimuli, with no changes in salty stimuli or in identification of bitterness. They also documented, for the first time, an increased sensitivity to fatty acids [25].

The molecular and hormonal mechanisms underlying these changes in taste perception are not completely elucidated. Recent studies demonstrate that food preferences are strongly affected by genetic factors, which contribute to individual taste variability [6], and that changes in macronutrient intakes may modulate the formation of bioactive lipids, influencing the eating behavior and energy metabolism by modifying the endocannabinoid and Peroxisome proliferator-activated receptors (PPAR) systems [41]. Changes in food preferences may be directly reflected in neuronal circuits in those brain areas involved in food rewarding and aversive activities. These factors along with hormone variations, particularly those secreted in the gastro-intestinal (GI) tract, may positively modify food preferences and energy metabolism. Therefore, further studies are needed to evaluate changes of taste sensitivity in patients undergoing SG in relation to circulating levels of endocannabinoids and the congeners PPAR alpha ligands, and to evaluate changes of circulating GI hormone profile and of neuronal circuits involved in taste perception. Concerning BS, a growing body of data indicate that weight loss induced by BS is only partially explained by mechanical alterations (restriction and/or malabsorption) and that important changes in the gut–brain axis occur after surgery, which may impact body weight loss. Indeed, a consistent increase in satiety hormones, such as the glucagon-like peptide 1 (GLP-1) and the peptide YY (PYY), has been described in patients operated on with GB [42,43]. Moreover, the increase in these hormones is associated with changes in appetite, food preference, and reduced brain-hedonic response to food [44,45]. The reduced hedonic impact of palatable food after GB has been confirmed by functional magnetic resonance imaging (fMRI) studies demonstrating weaker activation of the mesolimbic brain area [46]. Most of the published data refer to GB; limited information is available on the effects of SG [47]. Although SG is considered a “restrictive” procedure, it produces multiple changes in gut hormones [43,48]. It is known that the GI tract is involved in several aspects of eating behavior through neural and hormonal signals. It is known that the enteroendocrine cells, distributed along the entire alimentary canal, “sense” all major nutrients and, consequently, release hormones and paracrine factors informing the brain [49]. GI hormones include the incretins GLP-1 and gastric inhibitory polypeptide (GIP), satiation peptides, such as cholecystokinin (CCK), PYY, as well as orexigenic hormones, such as ghrelin. Recent evidence indicates that satiety hormones are also involved in the hedonic and emotional rapport with food, i.e., the food reward response [50].

Food preferences affected by genetic factors involved in taste variability and changes in macronutrient intakes may modulate the formation of bioactive lipids influencing eating behavior and energy metabolism by modifying the endocannabinoid and peroxisome proliferator-activated receptor (PPAR) systems, which may work to fine tune body metabolism in response to dietary exposure to taste stimuli. The endocannabinoid system regulates “on demand” the production and degradation, by specific pathways, of arachidonic acid derivatives, N-arachidonoylethanolamide (anandamide, AEA) and 2-arachidonoylglycerol (2-AG), and their high-affinity cannabinoid receptors (CB) 1 and CB 2 [50]. These receptors are widely expressed in peripheral tissues and in the central nervous system. The endocannabinoid system is known to play a crucial role in energy metabolism by influencing food intake and reward at the level of the hypothalamus and nucleus accumbens, respectively, as well as by modulating energy expenditure in peripheral tissues in experimental models and humans [51]. Moreover, it has been recently shown that the endocannabinoid system influences dietary fat sensitivity in both the oral cavity and intestine via CB1 receptors [52]; endocannabinoids also enhance hedonic eating [53]. These data suggest that the endocannabinoid system may regulate body energy storage and metabolism based on energy needs and genetic factors that influence taste sensitivity. Changes in circulating levels of endocannabinoids after BS have been described [54]; however, no association between eating pattern and taste sensitivity has been determined yet.

Gustatory stimulation by palatable foods induces dopamine release in the mesolimbic reward pathway connecting the ventral tegmental area (VTA) to the nucleus accumbens (NAc) within the ventral striatum. Sensory stimuli from taste buds are transmitted as signals through the solitary nucleus and in the upper medulla to other brainstem nuclei important for food intake and digestion [55]. This network may play a crucial role in linking sensory-hedonic experiences to the motivational components of reward, as well as emotionality, providing conscious awareness of these urges. Only recently, fMRI studies have demonstrated a spatially segregated and ordered chemotopic cortical representation of taste in the anterior insula [56]. This finding implies a functionally oriented organization of taste processing, which will be at the base of the discrimination between pleasant or unpleasant foods. As a consequence, bariatric nutritionist could play an additional key role [57] in monitoring patients’ taste changes during post-BS follow-up and in correcting patient’s food behavior on the basis of their taste, desire, and enjoyment.

We acknowledge some limitations, including the low number of patients and the lack of a control group. Furthermore, we are conscious that the assessment of the changes in taste perception and of the frequency of food intake by other techniques, such as constant stimuli, two-alternative forced choice, three-alternative forced choice, and Burghart taste strip test, could be more accurate. However, the obtained results on the changes in taste perception and on the frequency of food intake assessment by validated questionnaires appeared to be reliable and reproducible.

## 5. Conclusions

SG is characterized by a postoperative change in taste and preference for foods. However, further investigation is needed to clarify this issue. As a future prospective point of view, the molecular, hormonal, and central mechanisms underlying these changes in taste perception need to be further elucidated, as the characterization of changes in gustatory perception and liking, and the associated endocrine-metabolic-molecular variations, will allow identifying the mechanisms underlying dietary preferences and related metabolic changes characteristic of patients after SG. Furthermore, these data could identify new targets able to modify obesogenic eating behavior, opening up a novel personalized therapeutic approach to obesity.

## Data Availability

The data included in this manuscript derived from the University database. We are not authorized to share the data with third-party organizations. However, the corresponding author is available to provide any explanation to the Editor if requested.

## Supplementary Materials

The following supporting information can be downloaded at: https://www.mdpi.com/article/10.3390/nu14102060/s1, S1: Food Frequency Questionnaire; S2: Taste, Desire and Enjoyment Change Questionnaire (TDECQ).
