# Supplementary material for: Changes in Food Choice, Taste, Desire, and Enjoyment 1 Year after Sleeve Gastrectomy: A Prospective Study
Source: Nutrients. 2022 May 14;14(10):2060. doi: 10.3390/nu14102060 (PMC9145557; doi:10.3390/nu14102060)
Supplement: Supplementary file 1 [file nutrients-14-02060-s001.zip › S1_Food_Frequency_Questionnaire.pdf]

# FOOD FREQUENCY QUESTIONNAIRE

**This questionnaire asks for some background information about what you eat.**

**Your answers will be treated as strictly confidential and will be used only for medical research.**

**Please put a tick in the box to indicate how often, on average, you have eaten the amount of each food during the past year.**

## Section A: FOOD AND AMOUNTS

**A1.** **Meat and fish (beef, pork, lamb, chicken, oily, fish, fish fingers, shellfish)**

- Never or less than once/month ☐
- 1-3 per month ☐
- Once a week ☐
- 2-4 per week ☐
- 5-6 per week ☐
- Once a day ☐
- 2-3 per day ☐
- 4-5 per day ☐
- 6+ per day ☐

**A2.** **Bread and crackers (weight bread, brown bread, chees biscuits, cream cracker)**

- Never or less than once/month ☐
- 1-3 per month ☐
- Once a week ☐
- 2-4 per week ☐
- 5-6 per week ☐
- Once a day ☐
- 2-3 per day ☐
- 4-5 per day ☐

6+ per day ☐

**A3. Dairy products and fats (cheese, salad cream, butter)**

- Never or less than once/month ☐
- 1-3 per month ☐
- Once a week ☐
- 2-4 per week ☐
- 5-6 per week ☐
- Once a day ☐
- 2-3 per day ☐
- 4-5 per day ☐
- 6+ per day ☐

**A4. Sweets and snacks (sweet biscuits, chocolate, cakes, ice cream, peanuts)**

- Never or less than once/month ☐
- 1-3 per month ☐
- Once a week ☐
- 2-4 per week ☐
- 5-6 per week ☐
- Once a day ☐
- 2-3 per day ☐
- 4-5 per day ☐
- 6+ per day ☐

**A5. Vegetables and fruits (carrots, spinach, broccoli, tomatoes, apples, pears, oranges, bananas)**

- Never or less than once/month ☐
- 1-3 per month ☐
- Once a week ☐
- 2-4 per week ☐
- 5-6 per week ☐
- Once per day ☐
- 2-3 per day ☐

4-5 per day ☐

6+ per day ☐

**A6. Drinks (coffee, beer, wine, fizzy soft drink, fruit juice)**

Never or less than once/month ☐

1-3 per month ☐

Once a week ☐

2-4 per week ☐

5-6 per week ☐

Once a day ☐

2-3 per day ☐

4-5 per day ☐

6+ per day ☐
