# Supplementary material for: Changes in Food Choice, Taste, Desire, and Enjoyment 1 Year after Sleeve Gastrectomy: A Prospective Study
Source: Nutrients. 2022 May 14;14(10):2060. doi: 10.3390/nu14102060 (PMC9145557; doi:10.3390/nu14102060)
Supplement: Supplementary file 1 [file nutrients-14-02060-s001.zip › S2_TDECQ.pdf]

TASTE, DESIRE AND ENJOYMENT CHANGE QUESTIONNAIRE (TDECQ)

The questions below relate to the extent your tastes and desires for certain foods or liquids have changed or stayed the same since undergoing bariatric surgery. Please circle the number that best represents the extent of any changes in taste and the extent of change of desires/enjoyment for certain foods or liquids. *(foods refers to all types of foods and liquids)

Section A: TASTE CHANGE

The rating scale is as follows:

1 - Much weaker flavor / taste

2 - Weaker flavor / taste

3 - No change in strength of flavor / taste

4 - Stronger flavor / taste

5 - Much stronger flavor / taste

A1. I noticed that the taste of salty foods has changed

1 ☐

2 ☐

3 ☐

4 ☐

5 ☐

A2. I noticed that the taste of sugar / sweet foods has changed

1 ☐

2 ☐

3 ☐

4 ☐

5 ☐

A3. I noticed that the taste of fatty/oily foods has changed

1 ☐

2 ☐

3 ☐

4 ☐

5 ☐

A4. I noticed that taste of sour/tart foods has changed

1 ☐

2 ☐

3 ☐

4 ☐

5 ☐

A5. I noticed that the taste of savory foods has changed

1 ☐

2 ☐

3 ☐

4 ☐

5 ☐

A6. I noticed that the taste of spicy or piquant foods has changed

1 ☐

2 ☐

3 ☐

4 ☐

5 ☐

A7. I noticed that the taste of bitter foods has changed

1 ☐

2 ☐

3 ☐

4 ☐

5 ☐

A8. I noticed that foods and liquids have a metallic taste

Section B: DESIRE CHANGE

The rating scale is as follows:

1 - Much weaker desire

2 - Weaker desire

3 - No change in desire

4 - Stronger desire

5 - Much stronger desire

B1. I noticed that the desire for salty foods has changed

1 ☐
2 ☐
3 ☐
4 ☐
5 ☐

B2. I noticed that the desire for sugar/sweet foods has changed

1 ☐
2 ☐
3 ☐
4 ☐
5 ☐

B3. I noticed that the desire for fatty/oily foods has changed

1 ☐
2 ☐
3 ☐
4 ☐
5 ☐

B4. I noticed that the desire for sour/tart foods has changed

1 ☐
2 ☐
3 ☐

4 ☐

5 ☐

B5. **I noticed that the desire for spicy/piquant foods has changed**

1 ☐

2 ☐

3 ☐

4 ☐

5 ☐

B6. **I noticed that the desire for bitter foods has changed**

1 ☐

2 ☐

3 ☐

4 ☐

5 ☐

B7. **I noticed that the desire for metallic tasting foods has changed**

1 ☐

2 ☐

3 ☐

4 ☐

5 ☐

B8. **I noticed that the desire for savory foods has changed**

1 ☐

2 ☐

3 ☐

4 ☐

5 ☐

Section C: ENJOYMENT CHANGE

The rating scale is as follows:

1 - Much weaker enjoyment

2 - Weaker enjoyment

3 - No chamge in enjoyment

4 - Stronger enjoyment

5 - Much stronger enjoyment

C1. I noticed that the enjoyment of salty foods has changed

1 ☐
2 ☐
3 ☐
4 ☐
5 ☐

C2. I noticed that the enjoyment of sweet foods has changed

1 ☐
2 ☐
3 ☐
4 ☐
5 ☐

C3. I noticed that the enjoyment of fatty/oily foods has changed

1 ☐
2 ☐
3 ☐
4 ☐
5 ☐

C4. I noticed that the enjoyment of sour/tart foods has changed

1 ☐
2 ☐
3 ☐

4 ☐

5 ☐

C5. **I noticed that the enjoyment of spicy/piquant foods has changed**

1 ☐

2 ☐

3 ☐

4 ☐

5 ☐

C6. **I noticed that the enjoyment of bitter foods has changed**

1 ☐

2 ☐

3 ☐

4 ☐

5 ☐

C7. **I noticed that the enjoyment of savory foods has changed**

1 ☐

2 ☐

3 ☐

4 ☐

5 ☐

C8. **I noticed that the enjoyment of metallic tasting foods has changed**

1 ☐

2 ☐

3 ☐

4 ☐

5 ☐
